# Supplementary material for: Potential of Bacterial Inoculants to Mitigate Soil Compaction Effects on Gossypium hirsutum Growth
Source: Plants (Basel). 2025 Jun 16;14(12):1844. doi: 10.3390/plants14121844 (PMC12196767; doi:10.3390/plants14121844)
Supplement: Supplementary file 1 [file plants-14-01844-s001.zip › plants-3635636-supplementary.pdf]

Table S1. Analysis of the variance of Shoot Dry Matter (SDM, g).

Analysis of variance					
	DF	SS	MS	F	Pr > FC
**Block**	3	38.24	3	0.7951	0.51031
**Soil Compactation (SC)**	3	1005.31	6	20.9039	0.00000
**Bacterial Mix (BM)**	1	223.13	5	13.9191	0.00123
**SC x BM**	3	352.02	2	7.3198	0.00153
**Residuals**	21	336.64	4		
**Total**	31	1955.35	1		
**coefficient of variation (%)**	13.81%				

Degrees of Freedom (DF), Sum of Squares (SS), Mean Square (MS), F-value (F), and Probability associated with F (Pr > F), $p < 0.05$.

Table S2. Meaningful Interaction: Unfolding Interaction. Unfolding of soil compaction at each level of the bacterial mix.

Analysis of variance					
	DF	SS	MS	F	Pr > FC
**Block**	3	38.24	12.74	0.7951	0.51031
**Bacterial Mix (BM)**	1	223.13	223.13	13.9191	0.00120
**SC: BM with**	3	1124.78	374.92	23.3882	0.00000
**SC: BM without**	3	232.54	77.51	4.8355	0.01030
**Residuals**	21	336.64	16.03		
**Total**	31	1955.35	63.07		

Table S3. Meaningful Interaction: Unfolding Interaction. Unfolding of bacterial mix at each level of the soil compaction.

Analysis of variance					
	DF	SS	MS	F	Pr > FC
**Block**	3	38.24	12.7461	0.7951	0.51031
**Soil Compactation (SC)**	3	1005.31	335.1036	20.9039	0.00000
**BM: SC 65**	1	557.78	557.78	34.7946	0.00000
**BM: SC 75**	1	0.9112	0.9112	0.0568	0.8139
**BM: SC 85**	1	2.4200	2.4200	0.151	0.7015
**BM: SC 95**	1	14.0450	14.0450	0.8761	0.3599
**Residuals**	21	336.64	16.0306		
**Total**	31	1955.35	63.0758		

Table S4. Cotton root dry matter (g) under different levels of soil compaction and inoculated with *Pantoea vagans* strain 7URP1-6 and *Exiguobacterium sibiricum* strain 19RP3L2-7.

Soil compactation	Bacterial mix	block	Cotton root dry matter (g)			
**(%)**	-	-	0-10 cm depth	10-20 cm depth	20-30 cm depth	30-40 cm depth
**65**	with	1	8.8	4.26	3.59	5.27
**65**	with	2	6.9	3.37	1.94	2.96
**65**	with	3	5.11	2.46	1.68	7.34
**65**	with	4	6	3	2	5
**65**	without	1	3.57	1.37	0.91	0.71
**65**	without	2	6.84	3.9	1.2	1
**65**	without	3	3.15	1.27	1.14	0.98
**65**	without	4	3	1.4	1	0.8
**75**	with	1	4.42	1.1	0.35	1.39
**75**	with	2	6.54	0.54	1.61	1.6
**75**	with	3	4.53	1.94	0.62	1.37
**75**	with	4	4	1.4	0.6	1.4
**75**	without	1	5.37	1.41	0.99	6.02
**75**	without	2	5.35	0.79	0.7	1.2
**75**	without	3	2.45	0.88	0.28	0.55
**75**	without	4	4	1	0.7	1
**85**	with	1	6.91	0.38	0.21	0.95
**85**	with	2	7.1	0.65	0.3	0.67
**85**	with	3	6.66	0.75	2.53	1.72
**85**	with	4	6	0.6	1	1
**85**	without	1	6.42	0.29	0.19	0.84
**85**	without	2	6.3	0.35	0.15	0.64
**85**	without	3	4.32	0.47	0.17	1.9
**85**	without	4	4	0.3	0.17	0.8
**95**	with	1	5.22	0.88	0.36	0.78
**95**	with	2	6.75	0.48	0.8	1.6
**95**	with	3	5.86	0.61	0.12	1.1
**95**	with	4	5	0.6	0.4	1
**95**	without	1	6.8	0.8	0.36	0.33
**95**	without	2	5.96	0.47	0.52	1.2
**95**	without	3	2.64	0.49	0.28	0.05
**95**	without	4	3	0.5	0.4	0.5
